# The Regulation of Astrocytic Glutamate Transporters in Health and Neurodegenerative Diseases

**DOI:** 10.3390/ijms21249607

**Published:** 2020-12-17

**Authors:** Alison C. Todd, Giles E. Hardingham

**Affiliations:** 1 UK Dementia Research Institute at the University of Edinburgh, Chancellor’s Building, Edinburgh Medical School, Edinburgh EH16 4SB, UK; Alison.Todd@ed.ac.uk; 2 Centre for Discovery Brain Sciences, University of Edinburgh, Hugh Robson Building, George Square, Edinburgh EH8 9XD, UK

**Keywords:** astrocyte, glutamate, excitotoxicity, neurodegenerative disease, EAAT1, EAAT2

## Abstract

The astrocytic glutamate transporters excitatory amino acid transporters 1 and 2 (EAAT1 and EAAT2) play a key role in nervous system function to maintain extracellular glutamate levels at low levels. In physiology, this is essential for the rapid uptake of synaptically released glutamate, maintaining the temporal fidelity of synaptic transmission. However, EAAT1/2 hypo-expression or hypo-function are implicated in several disorders, including epilepsy and neurodegenerative diseases, as well as being observed naturally with aging. This not only disrupts synaptic information transmission, but in extremis leads to extracellular glutamate accumulation and excitotoxicity. A key facet of EAAT1/2 expression in astrocytes is a requirement for signals from other brain cell types in order to maintain their expression. Recent evidence has shown a prominent role for contact-dependent neuron-to-astrocyte and/or endothelial cell-to-astrocyte Notch signalling for inducing and maintaining the expression of these astrocytic glutamate transporters. The relevance of this non-cell-autonomous dependence to age- and neurodegenerative disease-associated decline in astrocytic EAAT expression is discussed, plus the implications for disease progression and putative therapeutic strategies.

## 1. Introduction

Glutamate is the predominant excitatory neurotransmitter in the brain, activating post-synaptic ionotropic N-methyl-D-aspartate (NMDA) and α-amino-3-hydroxy-5-methyl-4-isoxazolepropionic acid (AMPA)/kainate receptors. Co-activation of these receptors allows the influx of Na^+^ ions, which depolarises the cell’s membrane potential, triggering an action potential. Once released, it is important that glutamate is rapidly cleared, since failure of this has two key consequences. Firstly, it will continue to stimulate the post-synaptic receptors after the initial signal has been sent, impairing the detection of the next signal that arrives—akin to a saturation phenomenon, and potentially leading to cell swelling due to ion influx [1]. Secondly, if this glutamate escapes from the synaptic zone it was released into it could activate unintended synapses, triggering activity where it should not. Significantly, if glutamate escapes the synaptic region it can activate extrasynaptic NMDA receptors: too much Ca^2+^ influx via these extrasynaptic NMDA receptors induces signalling cascades that initiate cell death programs [2,3]. 

**Figure 1:**
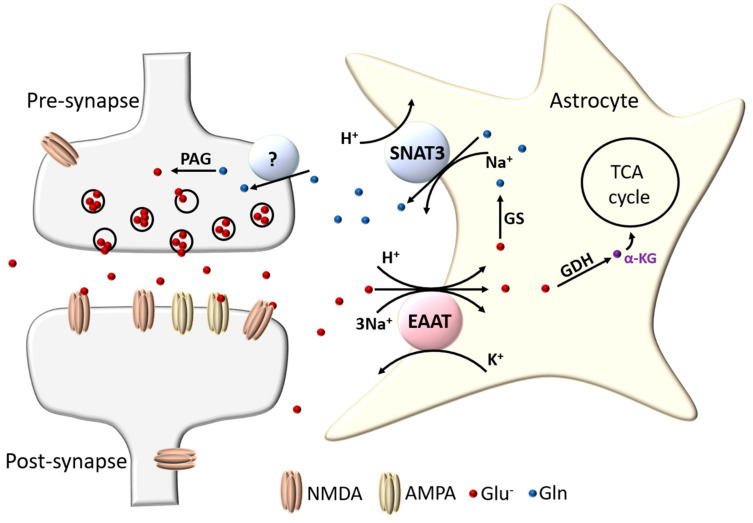
Glutamate-glutamine cycle. Glutamate (Glu^−^) released after excitatory transmission is collected by astrocytic EAAT transporters 1 and 2. The glutamate is then either converted into α-ketoglutarate (α-KG) via glutamate dehydrogenase (GDH) or transaminase reaction and enters the TCA cycle, or else is converted into glutamine (Gln) by glutamine synthetase (GS). Astrocytes excrete Gln back into the extracellular environment via the Na^+^ driven SNAT3 transporter, which is then taken up by an as yet unconfirmed neuronal Gln transporter. Neurons then convert Gln back to Glu^−^ via a phosphate-activated glutaminase (PAG) reaction to replenish their vesicular Glu^−^ stores.

Astrocytes have long been known to be important in promoting the survival of neurons and for counteracting the toxic effects of glutamate [4,5,6,7]. This protection is largely due to their ability to take glutamate up from the extracellular environment via transporters located on astrocytic membranes, preventing excitotoxicity and associated oxidative stress [8,9]. Once inside the astrocytes, glutamate is then either converted into α-ketoglutarate by glutamate dehydrogenase (GDH) or transaminases and shunted into the astrocytic TCA cycle, or else converted into glutamine by the enzyme glutamine synthetase (GS) [10,11]. Glutamine is not toxic to neurons, and is extruded by the SNAT3 glutamine transporter into the extrasynaptic space, which can then be taken up by neurons and converted back into glutamate via the neuronally expressed phosphate-activated glutaminase (PAG), thus replenishing pre-synaptic glutamate stores [12,13,14,15]. This glutamate recycling pathway is referred to as the glutamate-glutamine cycle (see Figure 1).

Astrocytic glutamate uptake and recycling is a vital part of CNS function. Key within this machinery are the two astrocytic glutamate transporters, excitatory amino acid transporters 1 and 2 (EAAT1 and EAAT2), that are responsible for the bulk of glutamate uptake. The astrocytic glutamate transporters EAAT1 and EAAT2 belong to the solute carrier 1A family of transporters (*SLC1A*), which includes two alanine serine cysteine transporters, ACST1 and ACST2, along with the five excitatory amino acid transporters, EAAT1-5 [16]. The EAATs are electrogenic secondary-active transporters, using the concentration gradients of their co- and counter-transported ions (Na^+^, H^+^, and K^+^) to drive the transport of glutamate against its concentration gradient into the cell [17]. Astrocytes are able to regulate any fluxes in their pH and membrane potential caused by this transport process through their high expression of membrane K^+^ channels (especially K_IR_4.1), their Na^+^/H^+^ exchangers and Na^+^/NCO_3_^−^ cotransporters, as well as dissipating charge and ionic changes throughout the astrocytic network via connexon coupling (see [18] for a recent review on astrocyte physiology). 

Unsurprisingly, reductions in the astrocytic glutamate transporters’ expression and function have been implicated in a number of CNS diseases, particularly epilepsy, along with several neurodegenerative diseases, as outlined below. It is therefore of interest to understand how these transporters are functionally regulated, as boosting their expression and function may offer a novel way to reduce severity and progression of neurodegenerative diseases. 

## 2. Glutamate Transporters in the Brain

Glutamate is found at high concentrations in the brain, at a concentration of approximately 10–14 mmol/L depending on region [19,20]. However, most of this glutamate is kept within intracellular compartments, with very low levels maintained in the extracellular fluid (around 3–4 μmol/L in the extracellular space of the hippocampus) [21,22]. Accordingly, there are numerous transporter proteins in the brain that are capable of facilitating glutamate transport to ensure that the right concentration of glutamate is maintained in the right compartment. These transporters fall into two broad categories: those which are found in intracellular compartments, such as the three vesicular glutamate transporters (vGLUT1-3) which package glutamate into synaptic vesicles, and those located on the plasma membrane of cells that can transport glutamate into (or out of) the cell [1,23,24]. The glutamate transporters that are found in the plasma membranes of brain cells consist of five sodium-dependent co-transporters, and one sodium-independent exchanger [17,25,26]. The sodium-independent exchanger, xCT, is found almost exclusively on astrocytes, but preferentially transports cysteine into the cell in exchange for extruding a glutamate molecule out of the astrocyte [27,28,29]. Due to the need to transport glutamate into cells against its electrochemical gradient, it is therefore the sodium-dependent class of transporters that are responsible for quickly sequestering extracellular glutamate back into cells. There are five known members of this family of transporters, excitatory amino acid transporters 1–5 (EAAT1-5). 

### 2.1. Excitatory Amino Acid Transporters

In the early 1970s, a high affinity sodium-dependent uptake system for the negatively charged amino acids l-glutamate and l-Aspartate was first described in synaptosomal preparations, which was hypothesised to be responsible for the accumulation of the putative excitatory neurotransmitter glutamate into cells [30,31]. A few years later Balcar and colleagues went on to show that this glutamate uptake system was also present in glial cells, but not until 1992 were EAATs first purified, with four independent groups cloning three distinct EAAT family members: Glt-1 (EAAT2), GLAST (EAAT1), and EAAC (EAAT3) [32,33,34,35,36]. The final two members were cloned in 1995 (EAAT4) and 1997 (EAAT5) [37,38]. 

All members of the EAAT family transport l-glutamate into cells under normal conditions using the electrochemical gradients of Na^+^ and K^+^ [1,17]. The EAAT family of transporters and the sodium-independent exchanger xCT both display high affinity for glutamate transport compared to the three vesicular glutamate transporters (K_M_ ≈ 2–100 μM for EAAT1-5 and K_M_ ≈ 20–55 μM for xCT versus K_M_ ≈ 1.5–3.5 mM for vGLUT1-3) [37,38,39,40,41,42,43,44,45,46,47,48,49,50]. Although the EAATs have relatively high affinity for glutamate, enabling them to sequester low concentrations of extracellular glutamate to prevent excitotoxicity, they interestingly have relatively slow transport cycle times (see Table 1). This problem may in part be overcome by rapid surface diffusion and transporter trafficking of the EAATs upon glutamate stimulation [51,52].

The different EAAT subtypes are found throughout the body, and within the brain they are found on different cell types and in different brain regions. Although they all transport glutamate into cells, each subtype possesses a different degree of chloride permeability, and it appears the function of each of these subtypes may vary. A summary of the five EAAT transporters is given in Table 1. Note well, for clarity in this review the transporters encoded by *SLC1A3/Slc1a3, SLC1A2/Slc1a2*, and *SLC1A1/Slc1a1* will be consistently referred to as EAAT1, EAAT2, and EAAT3, respectively, independent of species. Rodent EAAT1, EAAT2, and EAAT3 are typically referred to as GLAST (*Slc1a3)*, Glt-1 (*Slc1a2)*, and EAAC (*Slc1a1)* in other studies. However, there is high amino acid homology between human and rodent proteins, with 96% identity shared between human EAAT1 and rat GLAST, 95% identity between EAAT2 and rat Glt-1, and 92% identity between EAAT3 and rabbit EAAC1 [39].

### 2.2. Location of Excitatory Amino Acid Transporters

The two subtypes EAAT1 (i.e., GLAST) and EAAT2 (i.e., Glt-1) are referred to as the astrocytic glutamate transporters as they are the only EAAT subtypes expressed on astrocytes, where they are predominantly found on fine astrocytic processes opposed to glutamatergic synapses [53,54,55]. EAAT1 is found in astroglia (including the Bergmann and Müller glia) throughout the brain, on which they are exclusively expressed, and are the primary collectors of glutamate in both the cerebellum and retina (via Bergmann and Müller glia, respectively) [56,57,58,59,60,61,62]. EAAT2 is the primary glutamate transporter in all other brain regions, most prominently in the hippocampus and cortex [54,57]. The location of EAAT2 is less astrocyte-exclusive, with some evidence suggesting it is also found to a small degree in neurons, particularly in the hippocampus and retina (see Zhou and Danbolt, 2013 for discussion). Combined, EAAT1 and EAAT2 make up a significant proportion of the total protein in the brain, representing ~2.1% of protein in the molecular layer of the cerebellum, 1.6% in the hippocampal *stratum radiatum*, and 1% of protein in forebrain tissue, and are by far the most abundant EAAT subtypes in the CNS [56]. 

A more recent study using label-free quantitative (LFQ) tandem mass-spectrometry to survey the regional protein expression of postnatal human brain tissue has confirmed that EAAT1 and EAAT2 are abundantly expressed in all regions sampled: the cerebellar cortex (CBC), mediodorsal thalamic nucleus (MD), striatum (STR), amygdala (AMY), hippocampus (HIP), primary visual cortex (V1C), and the dorsal prefrontal cortex (DFC) [63]. Apart from the cerebellar cortex (where EAAT2 was within the 93rd percentile), EAAT2 was the most abundantly expressed of the transporters across all regions, sitting in the 97th (MD), 98th (STR, AMY, HIP, and DFC), and 99th (V1C) percentile of most abundant proteins within each region. EAAT1 on the other hand was more abundantly expressed in the CBC, being in the 99th percentile of expressed proteins, with consistently high, although lower than EAAT2, expression across other regions: 94th percentile in the AMY; 95th in MD, STR, and HIP; and 97th percentile in the V1C and DFC (calculated from supplementary data Table 10 of Carlyle et al., 2017) [63].

The EAAT3 subtype is exclusively neuronal, and is found on neurons throughout the brain [65,66]. It typically localises on dendritic spines and not axon terminals, with its highest expression seen in the hippocampus, at a concentration of 0.013 mg/g [65]. This is 100 times lower than EAAT2 levels in the same region, so even in the hippocampus it may only contribute modestly to glutamate clearance. Consistent with these earlier reports, the more recent LFQ mass spectrometry data showed a relatively low protein abundance of EAAT3 ranging from the 13th percentile in the V1C to the 26th percentile in the MD, and sitting at just the 21st percentile of abundance in the hippocampus (from supplementary Table 10, Carlyle et al., 2017) [63]. This is compared to EAAT1 and EAAT2 which were in the top percentiles of expressed proteins across all regions in these samples (>90th percentile for both EAAT1/2 in all regions) [63]. 

EAAT4 is another neuronally expressed glutamate transporter, however its expression profile is more restricted than that of EAAT3, being found primarily on Purkinje cells of the cerebellum, with some sparse expression in certain subregions of the forebrain and midbrain [67,68]. It represents about 0.2% of protein in the molecular layer of the cerebellum, which is approximately 10 times less than the predominant astroglial subtype in this region, EAAT1 (which represents 1.8% of total cerebellar protein), and similar to EAAT2 levels (0.3% of total protein) [56,67]. Again, consistent with these earlier findings, Carlyle and colleagues more recent mass spectrometry data revealed EAAT4 to be most abundantly expressed in the cerebellar cortex, in the 89th percentile of proteins. This is less than both EAAT1 and EAAT2 in this region, but significantly higher than EAAT4′s expression across all other regions (8th percentile in HIP, 10th in V1C, 14th in STR and DFC and 19th in MD and AMY), confirming that EAAT4 is primarily restricted to the cerebellum [63]. The final member of the family, EAAT5, has only been found in the eye, where it is located on synaptic terminals of retinal rod bipolar cells as well as rod and cone photoreceptors [38,69]. Again, the astrocytic EAAT1 subtype is expressed more strongly in the retina (located on Müller glia) than EAAT5, and there is evidence that EAAT5 may physiologically act as a chloride channel rather than a glutamate transporter in these retinal neurons [70,71].

### 2.3. Structure and Function of Excitatory Amino Acid Transporters—The Case for Astrocytic EAAT1 and EAAT2

Given that the glutamate concentration within cells is over 1000-fold higher than that in the extracellular space (≈10 mmol/L compared to 4 μmol/L), combined with the fact that glutamate is an anion carrying −1 charge, transporters responsible for sequestering glutamate must overcome both concentration- and electrochemical gradients. All EAATs support the transport of l-glutamate as well as d and l-Aspartate, displaying a relatively high affinity for inward l-glutamate transport, with reported K_M_ for glutamate ranging from 10–100 μM (see Table 1) [38,39,72]. This high affinity allows the transporters to continue to work efficiently to uptake glutamate under the low concentrations of the seen in the extracellular space. However, the times to complete one transport cycle of a glutamate molecule for the EAATs are relatively low, from 10 ms for EAAT3, 60–70 ms for EAAT1 and 2, >100 ms for EAAT4 and >1000 ms for EAAT5 [40,41,43,44,45]. Although this may seem a limitation as glutamate is required to be quickly cleared, the transporters (especially EAAT1 and EAAT2) are some of the most abundantly expressed proteins in the CNS (see Table 1), and furthermore appear to undergo rapid surface diffusion and shuttling, helping to overcome transporter saturation and slow transport cycle times [51]. 

The mechanism of EAAT transport was debated for some time, but it has now been established that the EAATs combine the transport of 1 glutamate molecule with the co-transport of 3 Na^+^ and 1 H^+^, whilst counter-transporting 1 K^+^ [32,73,74,75]. This has been supported by more recent studies using the homologous archaeal glutamate transporters Glt_Ph_ and Glt_Tk_ whose structures were crystallised in 2004 and 2013 [76,77,78,79]. As a result of this stoichiometry, there is a net +2 charge per molecule of glutamate transported, facilitating the inward movement of the otherwise negatively charged glutamate by using the Na^+^ and K^+^ electrochemical gradients to drive transport into the cell. This stoichiometry is estimated to allow the internal glutamate concentration to be in the order of 10^6^ times greater than the external concentration under physiological conditions, ensuring the transporters work to take up rather than extrude glutamate under normal conditions where the concentration differential is only in the order of 10^3^ [1,19,20,22]. The electrogenic nature of EAAT function also enables their function to be measured by both patch clamp and two-electrode voltage clamp electrophysiology [80,81,82]. Although this transporter stoichiometry is believed to be common to all members of the EAAT family, meaning all EAATs could help facilitate glutamate clearance, there is a difference between the EAATs. As well as functioning as a transporter, EAATs can also act as ligand-gated ion channels, with glutamate activation leading to an uncoupled conductance of Cl^−^ through the channel [37,40,41,83]. However, the level of anion conductance varies largely between the different subtypes [84]. EAAT4 and EAAT5 display the largest ion conductance, with their Cl^−^ conductance being greater than that of their glutamate uptake, EAAT1 and EAAT3 have intermediate ion conductance, while EAAT2 displays very little conductance at all [26,37,38,41]. Recent work has suggested that EAAT5 uses this anionic conductance to act as an “inhibitory” glutamate receptor in retinal cells, hyperpolarising these cells’ membrane potentials following glutamate activation [71,85,86]. It is likely that both the EAAT4 and EAAT5 subtypes do not physiologically function as glutamate uptake systems, but instead act as Cl^−^ channels. 

The first support for astrocytic glutamate transporters EAAT1 and EAAT2 being primarily responsible for glutamate clearance rather than neuronal subtypes came from studies using autoradiographic localization, which found the bulk of cleared glutamate was seen in glial cells [87,88]. Electrophysiological recordings later went on to show that this astrocytic glutamate clearance was mediated by EAAT1 and EAAT2 [89,90]. Final evidence that it is the astrocytic EAAT1 and EAAT2 subtypes, and not the neuronal EAAT3, that are responsible for glutamate clearance comes from knockout studies. 

An EAAT2 knockout animal was generated in 1997, which developed lethal seizures resulting in an 80% death rate by 13 weeks of age, compared to 100% survival in controls [91]. It was found that there was a slower clearance of synaptically released glutamate in knockout animals, with neuronal degeneration appearing specifically in the hippocampal CA1 region [91]. In 1998, the group went on to generate an EAAT1 knockout animal [92]. Unlike the EAAT2 knockout, removal of EAAT1 did not appear to be lethal, and brain development appeared normal. The group focused on the cerebellum, given that is EAAT1′s prominent region of expression, and found that in EAAT1 knockouts glutamate uptake in this region was nearly half that of wild-types. Although finding no difference in basic motor tasks, they found a significant impairment in the knockouts’ ability to complete a more challenging rotor-rod experiment. Furthermore, they found that mutant EAAT1 animals, but not wild-types, were susceptible to cerebellar edema following cold injury [92]. Inevitably, in 2006 the group reported on a double EAAT1/EAAT2 knockout animal. Unlike the single mutants, the double knockout of EAAT1 and EAAT2 was embryonic lethal, with mice dying by E17-18, and brain-wide abnormalities in structure observed [93]. These studies highlight the vital importance of these transporters in the CNS.

In 1997, a second group generated a mouse knockout for the neuronal glutamate uptake transporter, EAAT3 [94]. Contrary to the neurological deficits seen in EAAT1 or EAAT2 knockout animals, removal of EAAT3 had no negative effect on brain formation or function over a period of >12 months. There was no impairment in motor skills, nor in memory, nor in susceptibility to induced seizures [94]. A limitation of all these studies, particularly for the EAAT2 knockout, is that they utilised global knockout models, and not astrocyte specific. As EAAT2 is reportedly expressed on neurons, this does not rule out neuronal EAAT2 glutamate uptake as being an important source of glutamate clearance to prevent excitotoxicity. Addressing this limitation, Rosenberg and colleagues produced conditional neuronal and conditional astrocytic EAAT2 knockout lines [95]. Whilst neuronal knockouts showed no difference in growth and lifespan, astrocytic EAAT2 mutants had lower weight gain and significantly higher mortality rates compared to controls [95]. In proteo-liposome preparations from forebrains they found glutamate uptake in astrocytic mutants was 25% of that in controls, whereas there was no difference in glutamate uptake in these preparations between neuronal knockouts and controls. EEG recordings further showed astrocytic EAAT2 knockouts to have significantly more seizure events than controls, with no difference between the conditional neuronal knockouts and controls [95]. 

Altogether, the evidence shows that it is EAAT1 and EAAT2 expressed on astrocytes that are primarily responsible for clearing extracellular glutamate to prevent excitotoxicity. The stoichiometry of the EAAT transporters provides one explanation for why astrocytes and not neurons are primarily responsible for glutamate homeostasis: uptake can result in significant depolarisation (up to 2+ charge per molecule). If neurons were required to take up the bulk of released glutamate, this process in itself would cause significant neuronal depolarisation, potentially leading to a hyper-excitable feedback loop. Additionally, uptake would result in a significant increase in internal Na^+^ concentration, which is counterproductive to the neuron’s need to remove internal Na^+^ following an action potential and could represent a metabolic strain on the neurons and impede their ability to sustain action potential firing.

## 3. Astrocytic EAAT Regulation

Before the different EAAT isoforms were isolated, it was observed that culturing cerebellar astrocytes in the presence of cortical neuronal conditioned media increased glutamate uptake [96]. Over a decade later, astrocytic EAAT1 and EAAT2 were first found to be significantly downregulated in the striatum following glutamatergic denervation, and the following year it was reported that EAAT1 was upregulated in cortical astrocyte cultures following activation of astrocytic AMPA and kainate receptors [97,98]. These reports implicated a role for neurons and neuronal activity in regulating astrocytic glutamate transporters. Following on from this work, Swanson and colleagues cultured cortical astrocytes alone or in the presence of cortical neurons, finding that isolated astrocytes expressed very low levels of EAAT1 and EAAT2, that was robustly induced by neuronal co-culture [99]. It was further documented by Gegelashvili and colleagues that physical culture of neurons with cortical astrocytes increased astrocytic EAAT1 expression, as well as inducing EAAT2 expression [100]. Additionally, the group showed that feeding pure cortical astrocytes with neuronal conditioned media was able to induce EAAT2 expression in astrocytes, although they did not find EAAT1 to be affected by conditioned media. They concluded from this work that a neuronally released soluble factor was responsible for the regulation of EAAT2, whereas contact mediated interactions were predominant in the regulation of EAAT1 [100]. Interestingly, this group had earlier reported that glutamate was able to increase cortical astrocytic EAAT1 expression, suggesting soluble factors could play a role in regulating this transporter as well [98]. Work since then has focused on discovering the signalling molecules and pathways behind this neuronal regulation (see Table 2 for overview, references: Hardingham lab unpublished data, [97,98,99,100,101,102,103,104,105,106,107,108,109]).

### 3.1. Regulation of EAAT Expression by Soluble Factors

#### 3.1.1. Cyclic AMP Signalling

One of the first chemicals shown to induce glutamate transporter function in astrocytes was the cyclic AMP analogue, dibutyryl cyclic AMP (db-cAMP) [110]. Primary astrocytes grown alone showed little response to glutamate and appeared flat, whilst astrocytes fed with db-cAMP became more morphologically complex with significantly greater glutamate uptake [98,110,111]. As a result, many researchers began to treat astrocyte cultures with db-cAMP as standard practice, to make them more reminiscent of their in vivo counterparts. This suggests that one potential mechanism for the neuronal regulation of astrocytic glutamate transporters is through an induction of the astrocytic cAMP signalling pathway. 

Further evidence for a role of cAMP signalling in astrocytic EAAT regulation comes from studies showing that application of the adenyl cyclase activator forskolin, to stimulate cAMP production, to both astrocyte cultures and striatal homogenates is able to increase glutamate uptake [102,112]. The mechanism behind this cAMP induced upregulation is less clear, with one group finding inhibition of cAMP’s downstream target of protein kinase A (PKA) to be sufficient to block the effects of forskolin, whilst others found an effect of PKA inhibition in pure astrocyte cultures but no effect on astrocyte glutamate transporter function when grown in the presence of neurons [102,112]. The latter finding suggests that although PKA activation may upregulate EAAT1 and EAAT2 activity in astrocytes in the absence of neurons, in the presence of neurons this pathway is occluded by other mechanisms that are responsible for the observed neuronal regulation of astrocytic EAATs. Also unclear is what neuronally derived factor could be responsible for astrocyte cAMP elevation, since astrocytes express many adenylate cyclase-activating Gs-coupled receptors. Indeed, different ones may play a role in different circumstances.

#### 3.1.2. Glutamatergic Signalling and EAAT Trafficking

Despite early reporting that astrocytic AMPA and kainate receptor activation may upregulate EAAT expression, and that denervation decreases EAAT2 expression, there still lacks a consensus as to whether glutamatergic synaptic activity has a role in astrocytic EAAT expression. Both work from our lab and others have found no effect of pharmacological blockade of neuronal activity on astrocytic EAAT expression [101,102]. Contrary to these findings, it has been reported that in hippocampal astrocyte-neuron co-cultures pharmacological block of synaptic activity does reduce protein levels of both EAAT1 and EAAT2 [113]. Furthermore, acute kainate injections to induce seizure activity in rats were seen to initially cause a significant increase in cortical EAAT2 expression, peaking after 4 h, before ultimately decreasing below baseline levels (following neuronal death) [114]. 

Although glutamatergic signalling’s role in regulating EAAT expression is not yet clear, it has been observed that glutamate treatment can increase functional astrocytic glutamate clearance, and that this increase in function is mediated directly by the activation of the astrocytic glutamate transporters, rather than glutamate receptors [115]. The mechanism for this glutamate mediated increase in EAAT function was found to be due to an induction of EAAT1 surface expression in mouse astrocytic cultures, with no change in total transporter protein expression [115]. More recently, it has been reported by two groups using quantum dot tracking that glutamate treatment increases astrocytic glutamate clearance via robustly increasing the surface diffusion of EAAT2 in rodent astrocytes from primary culture, organotypic hippocampal slice and acute slice preparations [51,52]. This increase in diffusion, especially in the synaptic region, leads to a faster turnover of unoccupied transporters thereby enabling more efficient clearance of the perisynaptic glutamate. Indeed, immobilising the diffusion of EAAT2 was found to alter synaptic kinetics, increasing both the rise and decay time of spontaneous excitatory postsynaptic currents, although total glutamate clearance was unaltered [51]. As well as surface diffusion, EAATs also undergo surface trafficking, with glutamate triggering Ca^+^-dependent internalization of EAAT2 through endocytosis [116,117]. On the other hand, surface expression of EAAT1 has been shown to be increased by glutamate application, as well as insulin-like growth factor through phosphatidylinositol-3-kinase signalling, whereas the ubiquitin ligase neuronal developmentally downregulated gene 4, isoform 2 (Nedd4-2) has been shown to decrease EAAT1 surface expression [115,118,119]. These dynamic mechanisms for regulating EAAT surface expression are likely to have a significant impact on the functional glutamate clearance speed and capacity of astrocytes, especially given the EAATs rather slow transport cycle times.

#### 3.1.3. Other Secreted Signals

The EAAT2 transporter is able to be regulated by neuronal secreted factors, and much of the work investigating EAAT regulation has been focused on EAAT2 in particular [100]. Epidermal growth factor application has been shown to upregulate EAAT2 expression through activation of NF-κB signalling, with neuron-dependent induction of astrocytic NF-κB having been shown to upregulate astrocytic EAAT2 expression [103,104,105]. Additionally, enhanced expression of Pax6, a well characterized transcription factor in astrocytes and the CNS [120,121], in pure astrocyte cultures has been recently shown to induce EAAT2 expression, while knockdown of Pax6 in astrocytes grown with neurons was seen to strongly repress neuron-induced EAAT2 expression [106]. However, the authors do not speculate upon the neuronally released factor(s) that may modulate EAAT2 expression through astrocytic Pax6. 

### 3.2. Contact Dependent Regulation: Notch Signalling

In contrast to neuronally released factors, relatively little work had investigated the role of contact-dependent signalling pathways in neuronal control of astrocytic EAAT1 and/or EAAT2 expression. It has been reasonably well established that unlike EAAT2, neuronal upregulation of EAAT1 expression is via a contact-dependent mechanism and not through a soluble factor, but the mechanism remained unclear [122]. Furthermore, it had not been established if contact-dependent signalling also has a role in EAAT2 regulation. 

**Figure 2:**
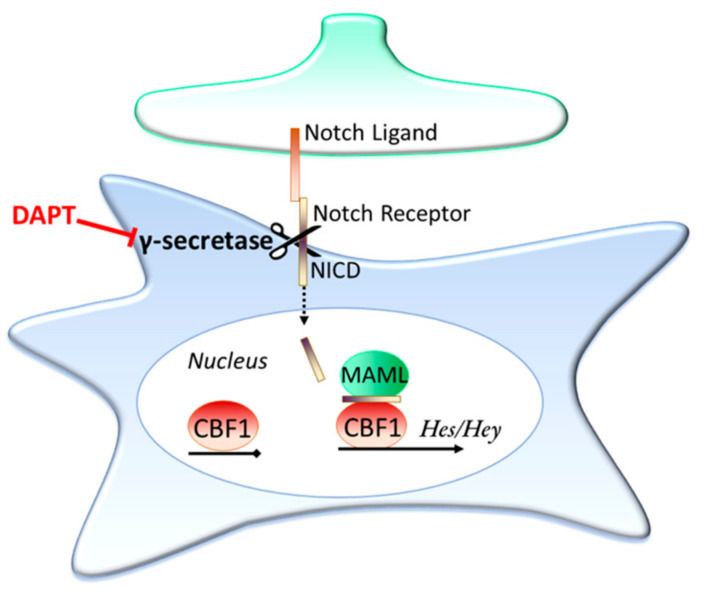
The Notch signalling pathway. Notch is a contact dependent signalling pathway. When a Notch ligand contacts a Notch receptor this initiates a cleavage event through the enzyme γ-secretase, releasing the Notch intracellular domain (NICD). The NICD then translocates into the cell nucleus, where it pulls down various proteins, such as MAML, and associates with the Notch effector, CBF1. This association turns on transcription, with the *Hes* and *Hey* family of genes prominent examples of genes transcribed by this cascade. The γ-secretase inhibitor DAPT is able to prevent activation of the Notch signalling pathway as the NICD is unable to be cleaved.

Notch is an important contact dependent signalling pathway present in astrocytes; in fact, it is the interaction of Notch ligands expressed on neuronally committed precursor cells with uncommitted precursors that first initiates the precursors’ development into astrocyte lineage cells [123]. An overview of the Notch signalling pathway is shown in Figure 2. Briefly, when Notch ligands (for example Delta and Jagged1 & 2) contact the receptors (Notch1-4) the receptors undergo cleavage by the enzyme γ-secretase, releasing the Notch intracellular domain (NICD) of the receptor. The NICD then translocates into the cell nucleus, where it associates with the Notch effector (CBF1) and Mastermind-like (MAML) to activate transcription, with the *Hes* and *Hey* family of genes being well-established examples of NICD/CBF1 target genes [124,125].

In drosophila only the EAAT1 subtype of high affinity glutamate transporters are found, where it is located on glia cells. Using this model system it was observed that Notch signalling mediated by neuronally expressed Delta ligands induced the expression of EAAT1 in glia cells [107]. If this is a conserved process, these results could suggest a role for Notch signalling not only in allowing astrocyte cell type differentiation, but also in inducing astrocytic EAAT expression. 

Strengthening the case for Notch, our lab recently demonstrated using a mixed-species culture model that neuron-to-astrocyte Notch signalling was a major regulator of astrocytic *Slc1a2* (EAAT2) and *Slc1a3* (EAAT1) expression, functionally boosting glutamate transporter activity in mouse astrocytes [101]. We also found, by expressing a constitutively active form of CBF1, that driving canonical Notch signaling is sufficient to induce glutamate uptake capacity in astrocytes [101].

Around the same time, another group found that endothelial cells were likewise able to induce EAAT1 and EAAT2 expression in mouse astrocytes through contact dependent Notch signalling [108]. The group has now gone on to show that the endothelial Notch ligands responsible for inducing the astrocytic Notch signalling pathway, and downstream increases in astrocytic *Slc1a2* and *Slc1a3* expression, are the two Delta-like Notch ligands, Dll1 and Dll4 [109]. 

Interestingly, a link between cAMP signalling and Notch in astrocytes has been reported [126]. Application of db-cAMP was observed to increase the amount of NICD that translocated into the cell nucleus, and that either application of the γ-secretase inhibitor DAPT to block NICD cleavage, or application of the PKA inhibitor H89 to prevent cAMP mediated PKA signalling, was sufficient to prevent this cAMP induced increase [126]. They confirmed that db-cAMP was able to induce Notch transcription, first by showing increased CBF1 activity via a luciferase assay. They then demonstrated that db-cAMP treatment increased both *Hes5* gene and Hes5 protein expression, which was also prevented by inhibition of either Notch (via DAPT application) or PKA (via H89) signalling [126]. This suggests the possibility that neuron contact-dependent and -independent signaling may converge on the Notch pathway, with neuron secreted factors that boost cAMP signaling, potentiating Notch signaling.

#### Is Ongoing Notch Signalling Required to Maintain Glutamate Uptake Capacity?

Having found that Notch signalling was required to functionally increase the activity of the astrocytic glutamate transporters, a key conceptual question remained for us: is this signalling required to maintain expression throughout development, or is EAAT expression set after their initial induction? If constant Notch signalling is required to maintain EAAT activity, then faulty neuronal Notch signalling could be a cause of impaired astrocytic glutamate clearance and increased excitotoxicity.

To investigate this possibility, we set up a mature coculture paradigm, growing established mouse astrocytes in the absence (monoculture, MC) or presence (coculture, CC) of rat neurons for 24 days (to neuron DIV24). The γ-secretase inhibitor DAPT was then applied to some cocultured cells from the point of neuronal plate down (DIV0) or else after two weeks of astrocyte-neuron coculture (DIV14) to block Notch signalling either from the outset or after the establishment of functional astrocytic glutamate transporters had occurred. Astrocyte glutamate transporter function was assessed by electrophysiological recording of astrocytic EAAT currents both on the 13th day of coculture with neurons in untreated astrocytes to confirm EAAT functional establishment, and then again on DIV24 across all conditions.

**Figure 3:**
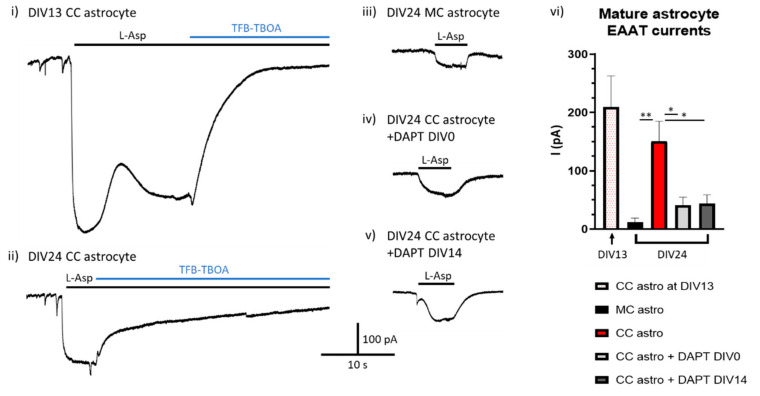
Notch signalling is needed to maintain astrocytic EAAT function. Example traces of EAAT mediated currents in response to 200 μM L-Asp in (**i**) DIV13 neuronal cocultured (CC) astrocyte, (**ii**) DIV24 CC astrocyte, (**iii**) DIV24 monocultured (MC) astrocyte, (**iv**) DIV24 CC astrocyte +DAPT from DIV0, and (**v**) DIV24 astrocyte +DAPT from DIV14. Cells were voltage-clamped at −80 mV and all recordings were done in the presence of 100 μM AP-5. (**vi**) At DIV24 there was a significantly larger EAAT mediated response in control CC astrocytes compared to MC astrocytes (*p* = 0.009, LME ANOVE, df = 29), as well as CC astrocytes treated with DAPT from either DIV0 or DIV14 (*p* = 0.028 & 0.027, for DIV0 and DIV14 respectively, LME ANOVA, df = 29). There was no difference in EAAT response in CC astrocytes treated with DAPT from DIV0 or from DIV14 after currents had been established. Recordings were taken from cells across at least three independent culture batches. * *p* < 0.05, ** *p* < 0.001.

We found that after two weeks exposure to neurons (DIV13) there was robust induction of astrocytic glutamate transport activity in astrocytes (Figure 3i). As expected, by DIV24 there was significantly greater EAAT activity in astrocytes cultured in the presence of neurons compared to astrocytes cultured without neurons (Figure 3ii,iii). The transport activity of cocultured astrocytes that had Notch inhibited from the outset was (as expected) lower than untreated cocultured cells, although some residual transport activity was seen (Figure 3iv). Most significantly, the EAAT transport activity in cocultured astrocytes that had Notch signalling inhibited on DIV14 of coculture (i.e., after the induction of EAAT function) was indistinguishable from those cells who had Notch signalling inhibited from the outset (Figure 3v,vi, Hardingham lab unpublished data). These data support a model whereby continuous neuron-to-astrocyte Notch signalling is required in order to maintain astrocytic glutamate transporter activity. This is consistent with the observation that in astrocytes isolated ex vivo both EAAT1/2 expression and Notch target genes decline in culture compared to their levels immediately post-isolation [101,127].

### 3.3. Epigenetic Regulation of EAATs

There is a growing interest into the role of epigenetic regulation of glutamate transporter expression in astrocytes, in particular the regulation of EAAT2. Astrocytes express both a number of epigenetic ‘writers’—enzymes capable of DNA and histone protein modification usually through the processes of methylation or acetylation, and ‘erasers’—enzymes capable of removing the epigenetic marks—i.e., demethylation and deacetylation—allowing for the epigenetic control of astrocytic gene transcription [128,129,130,131,132,133]. With respect to the glutamate transporters, it has been shown that overexpression of the epigenetic ‘erasers’ histone deacetylase (HDAC) 1, 3, 6, and 7 all reduce EAAT2 promotor activity, which can be reversed by treatment with HDAC inhibitors [134]. There are suggestions that altered epigenetic regulation of EAAT expression may be a factor in various diseases. For example it has been observed that in tissue samples from malignant glioma tumors there is a near total absence of astrocytic EAAT2 expression, which was explained by the pronounced hypermethylation of the EAAT2 promotor, resulting in the abolishment of EAAT2 transcription [135,136]. Treatments to inhibit both DNA methyltransferase (an epigenetic ‘writer’ that causes epigenetic transcriptional repression) and HDAC (which likewise leads to transcriptional repression) were successfully able to increase astrocytic EAAT2 expression in these glioma cell lines [135]. These findings raise the possibility of targeting epigenetic mechanisms as a way of controlling astrocytic EAAT expression in the context of disease [137].

## 4. Astrocytic EAAT in Ageing and Neurodegenerative Disease

As noted above, disruption to glutamate homeostasis has the capacity to disturb synaptic transmission, and by extension, synaptic connectivity and synaptic plasticity of both the classical and homeostatic type. [138,139,140,141], although direct evidence so far extends to Hebbian, spike timing-dependent plasticity [142]. Given the importance of plasticity in shaping circuits in development, one can envisage that even mild disruption to astrocyte-mediated glutamate homeostasis may contribute to deficits in early life brain disorders. Indeed, EAAT2 variants have been associated with cerebral palsy in pre-term infants [143], and with grey matter deficits and working memory in schizophrenia, along with altered EAAT1 and EAAT2 mRNA expression seen in some CNS regions of schizophrenic patients [144,145,146]. Alterations in astrocytic EAAT expression have further been associated with attention deficit hyperactivity disorder [147,148], autism [147,149], and depressive illnesses [150,151,152]. Additionally, given the protective effects of transient, phasic synaptic NMDAR activation, [153,154], perturbations which interfere with this are likely to be deleterious. Moreover, aberrant glutamate homeostasis leads to toxic extrasynaptic NMDAR activation, implicated in the pathophysiology of a number of disorders [155], including stroke [156], epilepsy [157,158], Huntington’s disease [159,160], and Alzheimer’s disease [161,162,163,164]. Though pharmacological inhibition [165], altering synaptic/extrasynaptic signaling balance [166,167], or uncoupling extrasynaptic NMDARs from downstream cascades [168,169] can mitigate these effects, it is important to understand the situations that lead to glutamate dyshomeostasis in the first place. A number of accounts have linked astrocytic glutamate transporter dysfunction both to epilepsy and the neurodegenerative diseases, such as amyotrophic lateral sclerosis (ALS), multiple sclerosis, Alzheimer’s disease, and Parkinson’s disease (see [170] for a recent review of EAATs in diseases of the CNS). An overview of diseases and disorders that aberrant expression and function of the two astrocytic glutamate transporters have been associated with is provided in Table 3. Interestingly, there have been recent suggestions that decreases in astrocytic EAAT expression may also be a natural feature of ageing in humans.

### 4.1. Epilepsy and EAATs

The first demonstration that increased glutamate concentrations are a feature of epilepsy came from the results of an in vivo microdialysis investigation into the concentrations of GABA and glutamate in the hippocampi of epilepsy patients from 1989 to 1992 [211]. The investigators found that an increase in glutamate concentration appeared in the epileptogenic hippocampus approximately 1.5 min prior to seizure onset, but not in the contralateral hippocampus. At the onset of seizure glutamate levels became further elevated, with concentrations in glutamate also beginning to increase in the opposing hippocampus. Ten minutes post seizure, the non-epileptic hippocampus glutamate concentrations had returned to baseline, whereas the epileptic side had persistently elevated glutamate levels >15 min post seizure [211]. The patients went on to receive surgical resection of the epileptic hippocampus, with microscopy of the removed tissue revealing moderate to severe pyramidal neuron loss throughout the hippocampal tissue along with reactive gliosis [211].

Given the deleterious effects of seizure-induced glutamate elevation in epilepsy, later work investigated the potential role of the glutamate transporters. A reduction in astrocytic EAAT2 expression was found in patients with temporal lobe epilepsy (TLE) that went on to develop hippocampal sclerosis, but no change was found in EAAT2 expression in patients without neuronal loss [188,189]. More recently, it was reported that there was a decrease in both EAAT1 and EAAT2 in epileptic hippocampi of patients with intractable treatment-resistant TLE [190]. It is not known if reduced astrocytic glutamate transporter function initiates some epileptic disorders or exacerbates it, or even if it is simply an outcome of prolonged disease in humans. However, animal models have shown that removal of functional astrocytic EAAT2, and not neuronal EAAT3, is sufficient to cause lethal epilepsy, demonstrating the possible contribution of astrocytic glutamate transporter dysfunction or hypo-expression in the development of certain epileptic disorders [91,212].

### 4.2. Neurodegenerative Diseases and EAATs

An increasing amount of research has gone into investigating the role of astrocytes and their glutamate clearance in different neurodegenerative diseases, with some links to disease emerging. One example is in amyotrophic lateral sclerosis (ALS), a motor neuron disease characterised by progressive loss of motor neurons in the motor cortex, somatosensory cortex and spinal cord, with around 90% of cases occurring sporadically and 10% with familial linkage. From 1992, it was discovered that impaired glutamate uptake in motor regions was a feature of tissue samples from patients with sporadic ALS, and in 1995 that there was a pronounced reduction specifically in EAAT2 protein levels in these tissue samples [180,181]. Additionally, one patient with sporadic ALS was found to have a mutation in the *SLC1A2* gene that resulted in an EAAT2 protein with reduced glutamate transporter activity, suggesting EAAT2 dysfunction may cause some cases of disease [182]. Familial forms of ALS on the other hand were found to be associated with mutations in the superoxide dismutase gene (*SOD1*), with SOD1 mutant protein being shown to reduce functional EAAT2 protein levels in animal models by initiating the cleavage of EAAT2 by Caspase-3 [183,184,185,186]. Specific deletion of *Slc1a2* in the spinal cord of mice was recently shown to be sufficient to lead to motor neuron degeneration by the fifth month of the mice’s lives [187]. Also of note, motor neurons carrying the *C9ORF72* expansion are also vulnerable to glutamate excitotoxicity [213], underlying the importance of glutamate homeostasis in this disorder. Finally, in work with our collaborators we have found that in the P301S tauopathy model mouse [214] which results in motor neuron loss of the spinal cord, there is an approximate 35% decrease in *Slc1a2* expression in disease mice [101]. It is unclear if it is reduced EAAT function that leads to initial motor neuron death, or if it is the death of neurons that leads to the decrease in functional EAAT (perhaps due to reduced contact-dependent Notch signalling), although a combination of both may be responsible.

Glutamatergic excitotoxicity has been suggested to play a role in PD and other synucleinopathies, with elevated glutamate levels hypothesized as a potential trigger for dopaminergic cell death. Most studies into EAAT dysfunction in PD have focused on animal models, however one human study found that there was reduced glutamate uptake in platelets derived from PD patients [197]. Many rodent models of PD have shown decreases in both EAAT1 and EAAT2 function and expression, including in the PD mutation *TJ-1* mouse model, MPTP injection and 6-ODHA lesion models [195,196,198]. However, some animal models have seemingly found the opposite, including one recent study which found that application of pathological α-synuclein oligomers to mice in vivo caused an increase in EAAT1 and EAAT2 expression that persisted into the timepoint when Parkinsonian phenotypes occurred [194]. No functional data for glutamate uptake in this in vivo setting was provided, and it would be interesting for future studies to investigate glutamate transporter function in this scenario, as the increase in expression may not necessarily be accompanied by an increase in functional uptake. One other possibility for these incongruous results is that astrocytes initially upregulate EAATs in response to α-synuclein oligomers (or their effect on neuronal activity) to try and reduce the excitotoxic effects, but over time with disease this upregulation is not sufficient, leading to neuronal loss and eventual reductions in EAAT expression.

As with motor neuron disease, there is interest in the role that glutamate dysregulation may play in the progression of neurodegeneration in Alzheimer’s disease (AD) and other dementias. From the 1990s, it was observed that amyloid β, the main protein found in AD-associated plaques, reduced the function and expression of EAAT1 and EAAT2 in rat hippocampal and cortical astrocytes [171,172,173]. Studies of human tissue samples have found the aberrant expression of both the normally astrocyte-specific EAAT1 transporter and the enzyme glutamine synthetase in subsets of cortical pyramidal neurons of AD patients, suggesting a marked dysfunction in astrocyte glutamate metabolism [174,175]. Reduced expression of both EAAT1 and EAAT2 have further been observed in the hippocampi of patients with AD, alongside a significant decrease in glutamate transporter function in human AD cortices [176,177]. Altogether, accumulated evidence suggests that impaired astrocytic glutamate recycling in the hippocampus and cortex is a feature of dementias and may play a role in the pathological progression of these disorders.

### 4.3. Ageing and EAATs

It appears that there may be a natural decline in the expression of astrocytic EAAT transporters with age. From a dataset produced by Barres and colleagues in 2016, where they reported the gene expression in astrocytes purified from healthy human CNS tissue (subjects ranging from 8 to 63 years old), the mean expression of the astrocytic glutamate transporters *SLC1A2* and *SLC1A3* was approximately 35% lower in the six samples from subjects >40 years old compared to the six samples from subjects < 40 years old (see Table 4; data from Zhang et al., 2016, Supplementary Table S6 [129]). A decrease in astrocytic (EAAT1 and EAAT2), but not neuronal (EAAT3) protein has further been observed as a feature in aged rats compared to younger animals, along with a decrease in *Slc1a2* expression seen in aged animals [215,216,217]. Thus, reduction in glutamate uptake capacity in ageing may contribute to it being by far the most important risk factor in neurodegenerative diseases.

## 5. Notch Signalling in Ageing and Disease

If impaired glutamate clearance is a feature of age and disease, and Notch signalling is required to maintain astrocytic EAAT function, then could impaired Notch signalling also be a feature of disease and ageing? A key enzyme in the Notch signalling pathway is the γ-secretase enzyme complex, which is responsible for the cleavage of the Notch intracellular domain, a required step for the induction of the Notch transcriptional pathway. The γ-secretase enzyme complex is itself comprised of four membrane proteins: presenilin (*PSEN1* and *PSEN2*), nicastrin (*NCSTN*), anterior pharynx-defective 1 (*APH1A* and *APH1B*), and presenilin enhancer 2 (*PSENEN*) [218]. Impairments in γ-secretase function have long been associated with Alzheimer’s disease. As well as γ-secretase’s role in cleaving the NICD, it is also involved in cleavage of the amyloid precursor protein (APP); increased cleavage of APP into toxic Aβ fragments leads to the production of amyloid plaques, a characteristic feature of AD [219]. As it turns out, mutations that cause familial early onset AD have predominantly been found to be in the presenilin component of the γ-secretase complex, *PSEN1* and *PSEN2* [220,221]. These mutations alter the activity of γ-secretase and cause an increase in production of toxic Aβ fragments.

An early human AD drug trial was run on the broad spectrum γ-secretase inhibitor semagacestat, with the belief that preventing toxic Aβ and amyloid production would improve disease outcome. However, the opposite occurred: inhibiting γ-secretase worsened AD progression, with this worsening eventually attributed to the incidental inhibition of Notch signalling [222,223]. In the light of recent findings, it is a possibility that one of the consequences of Notch inhibition for the CNS was reduced glutamate uptake capacity. Of note, presenilin mutations that give rise to early onset AD and increased pathogenic Aβ production are concurrently found to have a significantly reduced ability to cleave the NICD, suggesting that although mutant presenilin might enhance production of toxic Aβ it also results in loss of Notch activation [224,225,226,227]. Again, this raised the possibility that reduced EAAT1/2 expression may be a feature of these early onset AD variants.

Finally, the same human dataset that saw a reduction in EAAT expression with age likewise found a significant reduction in Notch pathway genes (including *HES5* γ-secretase components) in aged human samples, as shown in Table 4 [129]. This raises the possibility that natural reductions in Notch signalling with age in humans could engender vulnerability towards reduced EAAT expression, increased excitotoxicity, and ultimately neurodegeneration.

## 6. Concluding Remarks

There is emerging evidence that there are reductions in both astrocytic Notch signalling and astrocytic glutamate transporter expression with age in humans, and that this may be further exacerbated in neurodegenerative disease [129,176]. As it appears that Notch signalling is required in order to maintain EAAT expression levels in astrocytes (albeit in a rodent model), it is unsurprising that the reductions in EAAT expression in ageing appear alongside reductions in Notch activity. It is also intuitive that there should be further reductions in both Notch signalling and EAAT expression in astrocytes in neurodegenerative disease, as loss of neurons will lead to loss of contact with astrocytes, and therefore loss of Notch signalling [101]. It is less clear whether this is a downstream symptom of disease, or whether disease can be the outcome of reduced Notch or EAAT function in astrocytes to begin with. Given mutations that alter the function of γ-secretase in such a way that reduces Notch signalling lead to familial AD it is certainly plausible that disease may in part be due to reduced EAAT function leading to excitotoxic synapse loss and neurodegeneration, particularly in patients where increased excitability and glutamate dysregulation are observed [228]. The failed results of the semagacestat drug trial, that worsened disease progression, certainly hint that blocking Notch signalling and perhaps reducing EAAT function exacerbate neurodegenerative disease [222,229].

An involvement of Notch signalling and astrocytic EAAT function would also provide a pleasing explanation of sporadic cases of AD in humans, and why dementia is not a feature of ageing in non-transgenic mice: as reductions in Notch signalling and EAAT are natural features of human (and not so far observed in rodent) ageing, this would mean the aged human brain is particularly vulnerable to the development of dementia, the time when sporadic dementias typically occur. An external factor, such as vascular deficits, reducing bioenergetic status of the brain and thus reduced capacity for energy-expensive glutamate uptake, may exacerbate progression in sporadic neurodegenerative disease where EAATs are under-expressed. A further possibility for the sporadic occurrence of AD and inter-patient variability in EAAT expression could be explained by differences in epigenetic repression of EAAT transcription between individuals. Whether astrocytic Notch, or the modulation of EAAT expression, could be manipulated in a specific enough way to avoid the myriad roles of Notch (or epigenetic regulation) plays elsewhere in the body may however be a major translational hurdle.

Several questions remain to be investigated. Firstly, although we have seen that Notch signalling from neurons to astrocytes is required to maintain EAAT function in a rodent cell model, it needs to be ascertained whether this is the case for human cells. Secondly, investigations into the potential involvement of impaired Notch signalling (and potentially by extension reduced glutamate clearance capacity) have effectively stalled following on from the findings that inhibiting the Notch effector in neurons did not lead to disease in mice [230,231]. Future studies are needed to determine whether impairing Notch in astrocytes contributes to disease, and what the downstream transcriptional results are, for example reduced *Slc1a2* and *Slc1a3* expression. Finally, although we agree that research into targeting γ-secretases as a treatment for AD should not be abandoned, perhaps chronic partial γ-secretase inhibition is also not the way forward for most cases of AD [222]. More promising are treatments that specifically inhibit γ-secretase’s cleavage of APP without altering γ-secretases ability to cleave the NICD, such as imatinib, although such treatments are still not answering the question of Notch and EAAT involvement in disease [232]. A novel way forward could be to instead investigate the effect of increasing astrocytic γ-secretase activity, or in the case of models based on γ-secretase mutations, specifically boosting Notch signalling in these cells, via direct or indirect mechanisms [233]. The ability to target drugs to specific cell types is still in its infancy (though see [234]), but once a cell type and target have been validated, greater attention and investment will invariably lead to results.

## 7. Materials and Methods 

### 7.1. Tissue Cultures and Stimulations

Astrocytes and neurons were cultured from E17.5 CD1 mouse and E20.5 Sprague Dawley rat embryos as previously described [235,236]. Cortices or spinal cords were dissected, enzymatically digested with papain and mechanically dissociated using a 5 mL pipette. Mouse cortical and spinal cord astrocytes were obtained by growing cells at low density in DMEM containing 10% fetal bovine serum and were passaged twice, using Trypsin (both Life Technologies), and plated onto coverslips in a 24-well plate. These astrocytes are >99% GFAP positive and <0.1% NeuN positive12. For mixed–species co-cultures, rat neurons were plated on top of a confluent layer of DIV14 mouse astrocytes and both astrocyte monocultures and astrocyte–neuron co-cultures were subsequently kept in Neurobasal-A medium containing B27 (both Life Technologies), but devoid of serum. Cultures were maintained up to DIV24, with regular feeding via 50% media exchanges conducted every three days. During this maintenance the cultured cells were either left untreated, treated with the γ-secretase inhibitor DAPT (10 μM) from DIV0, or treated with DAPT from DIV14.

### 7.2. Electrophysiological Recordings

Cultures were recorded from at either DIV13 (co-cultured astrocytes prior to DAPT application) or at DIV24 (all conditions), as described [101]. Coverslips containing cortical neurons and astrocytes were transferred to a recording chamber perfused (at a flow rate of 3–5 mL min^−1^) with an external recording solution composed of (in mM): 150 NaCl, 2.8 KCl, 10 HEPES, 2 CaCl_2_, 1 MgCl_2_, and 10 glucose, pH 7.3 (320–330 mOsm). Patch-pipettes were made from thick-walled borosilicate glass (Harvard Apparatus, Kent, UK), and when filled with the internal recording solution had tip resistances of 4–8 MΩ. A KCl-based internal was used, composed of (in mM): KCl 130, glucose 4, HEPES 10, EGTA 0.1, CaCl2 0.025, and sucrose 20; pH 7.2 with KOH. Astrocytes were voltage-clamped at −80 mV and any cells with a resting membrane potential >−60 mV upon break-in were discarded. To determine the maximal induced EAAT transport current, 200 μM l-Aspartate was bath applied followed by the addition of the high affinity EAAT inhibitor TFB-TBOA (20 μM) to ensure that any l-Aspartate-induced current was mediated by the transporter. AP5 (100 μM) was included in the external solution of all astrocyte recordings to block the activation of NMDARs by l-Aspartate. Recordings were at room temperature (21 ± 2 °C) using a Multiclamp 200B amplifier (Molecular Devices, Union City, CA, USA). Recordings were filtered at 5 kHz and digitized online at 20 kHz via a BNC-2090A/PCI-6251 DAQ board interface (National Instruments, Austin, TX, USA) and analysed using WinEDR 3.6 software (Dr John Dempster, University of Strathclyde, Glasgow, UK).

### 7.3. Statistical Analysis

Results are given as mean ± standard error of mean (SEM), unless otherwise stated. A linear mixed effects (LME) analysis of variance (ANOVA) model was run using R statistical software to assess significance of treatment conditions. This analysis was set up to take variation between the different cultures into consideration. Each data point was coded for the culture batch it was taken from, and the variation in results due to week by week culture variation was then incorporated as a random variable into the statistical analysis.

## Figures and Tables

**Table 1:** Excitatory amino acid transporter family overview.

Protein	Gene	Cl^−^ Conduct.	Kinetics	Location	Protein Abundance
EAAT1	*SLC1A3*	Mod	K_M_ = 22–48 μM Cycle time = 62 ms	Astrocytes (incl. Bergmann & Müller glia); Predominant EAAT subtype in cerebellum (1.8 mg/g of protein) and retina	99th percentile of protein found in human CBC; ≥95th in V1C, DFC, MD, STR and HIP; F794th in AMY
EAAT2	*SLC1A2*	Low	K_M_ = 25–97 μM Cycle time = 70 ms	Astrocytes (and some sparse neurons); Predominant EAAT subtype in hippocampus (1.3 mg/g of protein) and cortex (0.8 mg/g)	99th percentile of protein found in human V1C; ≥95th in DFC, HIP, AMY, STR and MD; 93rd in CBC
EAAT3	*SLC1A1*	Mod	K_M_ = 42–62 μM Cycle time = 10 ms	Neurons (typically on spines); Highest concentration in hippocampus (0.013 mg/g of protein)	26th percentile of protein found in human MD; 21st in HIP; ≤20th in AMY, STR, CBC, DFC and V1C
EAAT4	*SLC1A6*	High	K_M_ = 2.5 μM Cycle time >166 ms	Cerebellar Purkinje cells (0.2 mg/g of protein in cerebellar molecular layer)	89th percentile of protein in human CBC; 9th in MD and AMY; <15th in STR, DFC and HIP
EAAT5	*SLC1A7*	High	K_M_ = 61–62 μM Cycle time >1000 ms	Retina (rod photo receptors, bipolar cells)	

Abbreviations: CBC = cerebellar cortex; V1C = primary visual cortex; DFC = dorsal prefrontal cortex; MD = mediodorsal thalamic nucleus; STR = striatum; HIP = hippocampus and AMY = amygdala. See ref [64] for references.

**Table 2:** Regulation of astrocytic glutamate transporters.

Treatment	*Slc1a3*/EAAT1	*Slc1a2*/EAAT2	Species
Neuronal coculture	Increased expression	Robust induction of expression	Mouse and rat; in vitro
Neuronal Conditioned Media	No	Yes	Mouse; in vitro
cAMP	Increases expression and function	Robust increases in expression and function	Mouse; in vitro
Glutamate	Downregulated by glutamaterigic denervation; Upregulated by AMPA receptor activation	Downregulated by glutamaterigic denervation	Mouse and Rat; in situ
Epidermal growth factor/NF-κB	No	Increased expression. Overlap with NCM and cAMP pathway	Mouse, rat and human; in vitro
Pax6	No	Induces expression in pure astrocytes; knockdown represses neuron coculture induction	Mouse; in vitro
Notch (Neuron/endothelial cell to astrocyte contact dependant)	Increases expression; inhibition decreases expression	Increased expression; inhibition decreases expression	Mouse, rat and drosophila; in vitro and in vivo

**Table 3:** CNS diseases and disorders associated with astrocytic glutamate transporter dysfunction.

Disease	Transporter	Observed Association	Refs
Alzheimer’s Disease (AD)	EAAT1, EAAT2	EAAT1/2 function and expression reduced by amyloid β; Aberrant EAAT1 expression in AD patient neurons; Reduced function and expression of EAAT1 and 2 in hippocampal and cortical AD tissue	[171,172,173,174,175,176,177,178,179]
Amyotrophic lateral sclerosis (ALS)/motor neuron disease	EAAT2	Impaired Glu uptake in patients with sporadic ALS; Reduced EAAT2 protein in tissue from motor regions; One reported case of a patient with a mutation in *SLC1A2* causing reduced EAAT2 activity; Familial ALS with *SOD1* mutations expected to reduce functional EAAT2 protein; Deletion of *slc1a2* in mice spinal cord leads to motor neuron degeneration; Reduction in *slc1a2* in P301S tauopathy mouse model	[101,180,181,182,183,184,185,186,187]
Epilepsy/temporal lobe epilepsy (TLE)	EAAT1, EAAT2	Reduced EAAT2 in TLE patients with hippocampal sclerosis; Reduced EAAT1 & 2 in treatment resistant TLE patients; Mouse EAAT2 KO → lethal epilepsy	[91,188,189,190]
Multiple sclerosis (MS)	EAAT1, EAAT2	Increased EAAT1 and EAAT2 mRNA and protein in MS optic nerve, with increased glutamate uptake; Loss of EAAT1 and EAAT2 in areas surrounding cortical lesions of MS patients; In rat EAE model cortex, increased EAAT2 mRNA and protein, increased EAAT1 mRNA but *decreased* protein. In rat EAE model cerebellum, increased EAAT1 and EAAT2 mRNA, but decreased EAAT1 and EAAT2 protein.	[191,192,193]
Synucleinop-athies (including Parkinson’s disease -PD)	EAAT1, EAAT2	Increased EAAT1 and EAAT2 expression following injection of α-synuclein oligomers in mouse striatum; Decreased EAAT1 and EAAT2 in rat striatum following dopaminergic denervation via MPTP treatment or 6-ODHA induced lesion rat models; Reduced glutamate uptake in platelets from PD patients; PD-related mutation *DJ-1* mouse model showed reduced EAAT2 function	[194,195,196,197,198]
Huntington’s disease (HD)	EAAT2	Decrease in EAAT2 mRNA expression in neostriatum of HD patients, decrease corresponding to disease severity; Decease in EAAT2 mRNA and protein in mice expressing mutant huntingtin;	[199,200]
Schizophrenia (SCZ)	EAAT1, EAAT2	Increased mRNA expression of EAAT1 in Brodmann’s area (BA)9; Increased mRNA expression of EAAT1 and EAAT2 in BA10; Increased EAAT1 mRNA and decreased protein in post mortem SCZ CNS tissue; Clozapine (used to treat SCZ) decreased EAAT2 expression;	[145,146,201,202]
Major depressive disorder (MDD)	EAAT1, EAAT2	Reduced mRNA expression of EAAT1 and EAAT2 in anterior cingulate, dorsolateral prefrontal cortex, locus coeruleus and hippocampus of human MDD patients; Decreased protein in orbitofrontal cortex of MDD patients;	[150,151,152,203]
Autism	EAAT1,F7EAAT2	EAAT1 mRNA expression upregulated; Decreased functional EAAT2 in conditional Fmr1 KO mouse astrocytes (mouse model of fragile-X)	[149,204]
Attention deficit hyperactivity disorder (ADHD)	EAAT1	Duplication of *SLC1A3* gene observed in clinical case of ADHD; *SLC1A3* rs1049522 allele significantly associated with ADHD; Increased EAAT1 mRNA expression in cerebellar cortex	[147,148]
Chronic pain	EAAT2	Decreased EAAT2 mRNA in rostral ventromedial medulla and spinal cord in rodent chronic pain models; Administration of EAAT2 antagonist alleviates hyperalgesia in rats; Analgesic effects of valproic acid suggested to be due to increasing EAAT1 expression.	[205,206,207,208,209]
Episodic ataxia type 6	EAAT1	Caused by mutations in *SLC1A3* altering properties of EAAT1	[210]

**Table 4:** Notch pathway and astrocytic EAAT expression in human astrocytes with age. There is a reduction in gene expression for both astrocytic EAAT transporters and Notch pathway associated genes with age in astrocytes isolated from human tissue.

Gene	Mean Human Expression in <40 y.o. (FPKM)	Mean Human Expression in >40 y.o. (FPKM)	Relative Expression with Older Age
Notch genes			
*HES1*	9.31	6.57	0.71
*HES6*	2.91	1.28	0.44
*HES5*	3.47	0.92	0.26
*HEY2*	1.40	1.57	1.12
*HEY1*	10.43	9.76	0.94
*BCL2*	5.41	4.75	0.88
Total FPKM	32.93	24.84	0.75
Notch receptors			
*NOTCH2*	22.36	14.39	0.64
*NOTCH1*	0.84	0.41	0.49
*NOTCH3*	0.53	0.20	0.38
*NOTCH4*	0.11	0.13	1.22
Total FPKM	23.83	15.14	0.64
γ-secretase genes			
*PSEN1*	7.87	6.99	0.89
*PSEN2*	1.23	0.67	0.55
*NCSTN*	24.59	11.58	0.47
*APH1A*	1.63	1.27	0.78
*APH1B*	5.07	4.72	0.93
*PSENEN*	0.26	0.65	2.51
Total FPKM	40.64	25.89	0.64
Notch effectors/activators			
*MAML1*	1.83	0.90	0.49
*MED8*	4.42	2.77	0.63
*RBPJ*	6.52	7.22	1.11
*FURIN*	0.46	0.17	0.38
Total FPKM	13.23	11.07	0.84
Sum Notch related genes (FPKM)	110.64	76.93	0.70
Glutamate transporters			
*SLC1A2*	2454.47	1521.31	0.62
*SLC1A3*	1146.57	797.77	0.70
Total EAAT (FPKM)	3601.05	2319.07	0.64
